# Vascular Smooth Muscle Cells Phenotypic Switching in Cardiovascular Diseases

**DOI:** 10.3390/cells11244060

**Published:** 2022-12-15

**Authors:** Hao-Yue Tang, Ai-Qun Chen, Huan Zhang, Xiao-Fei Gao, Xiang-Quan Kong, Jun-Jie Zhang

**Affiliations:** 1 Department of Cardiology, Nanjing First Hospital, Nanjing Medical University, No. 68 Changle Road, Nanjing 210006, China; 2 Department of Cardiology, Nanjing Heart Centre, No. 68 Changle Road, Nanjing 210006, China

**Keywords:** vascular smooth muscle cell, atherosclerosis, neointimal hyperplasia, aortic aneurysms, vascular calcification

## Abstract

Vascular smooth muscle cells (VSMCs), the major cell type in the arterial vessel wall, have a contractile phenotype that maintains the normal vessel structure and function under physiological conditions. In response to stress or vascular injury, contractile VSMCs can switch to a less differentiated state (synthetic phenotype) to acquire the proliferative, migratory, and synthetic capabilities for tissue reparation. Imbalances in VSMCs phenotypic switching can result in a variety of cardiovascular diseases, including atherosclerosis, in-stent restenosis, aortic aneurysms, and vascular calcification. It is very important to identify the molecular mechanisms regulating VSMCs phenotypic switching to prevent and treat cardiovascular diseases with high morbidity and mortality. However, the key molecular mechanisms and signaling pathways participating in VSMCs phenotypic switching have still not been fully elucidated despite long-term efforts by cardiovascular researchers. In this review, we provide an updated summary of the recent studies and systematic knowledge of VSMCs phenotypic switching in atherosclerosis, in-stent restenosis, aortic aneurysms, and vascular calcification, which may help guide future research and provide novel insights into the prevention and treatment of related diseases.

## 1. Vascular Smooth Muscle Cells

As the fundamental formative and functional constituent of the arterial wall, vascular smooth muscle cells (VSMCs) play a role in maintaining vascular tone and integrity, adjusting the pressure in the lumen, distributing the blood volume, and contributing substantially to preserving vascular homeostasis [1,2]. In an internal homeostatic environment, highly differentiated VSMCs in the tunica media are spindle-shaped, express abundant contractile proteins (e.g., α-smooth muscle actin (α-SMA), calponin 1 (CNN1), smooth muscle myosin heavy chain (SMMHC), and smooth muscle protein 22-α(SM22α)), and undergo negligible proliferation and migration [3,4,5].

**Figure 1:**
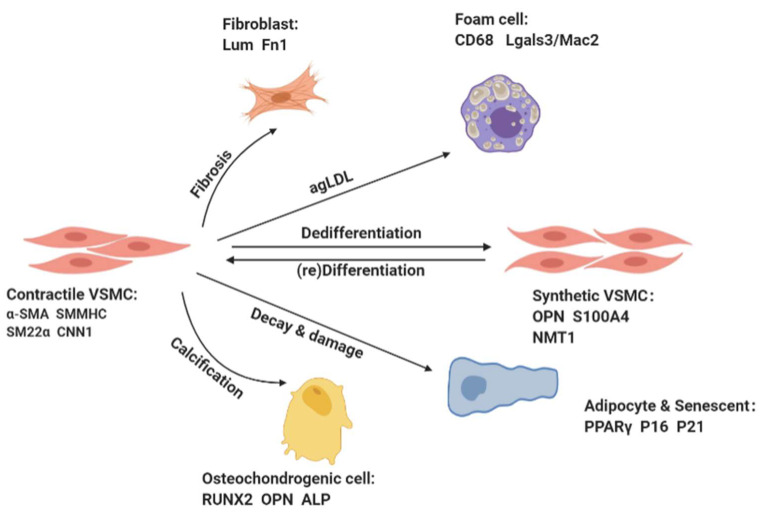
VSMCs can undergo reversible phenotypic switching in response to stimulation by various factors. The differentiated VSMCs in the tunica media are spindle-shaped, expressing abundant contractile proteins (e.g., α-smooth muscle actin (α-SMA), calponin 1 (CNN1), smooth muscle myosin heavy chain (SMMHC), and smooth muscle protein 22-α(SM22α)), and undergo negligible proliferation and migration. The dedifferentiated synthetic VSMCs are epithelioid-shaped, expressing decreased levels of contractile proteins and increased levels of synthetic proteins (e.g., osteopontin (OPN), vimentin, S100 calcium-binding protein A4(S100A4), and N-myristoyltransferase 1 (NMT1)), and they exhibit a high proliferation and migration capacity and synthesize massive amounts of extracellular matrix (ECM). VSMCs can also acquire the characteristics of other cell types, such as osteoblasts, fibroblasts, and foam cells.

VSMCs primarily show poor proliferation and synthesis ability, but unlike osteoblasts and cardiomyocytes that are end-differentiated, VSMCs exhibit notable plasticity [6]. Stimulating or damaging factors in the cellular environment, such as vascular wall injury, growth factors (e.g., platelet-derived growth factor BB (PDGF-BB)) and tumor necrosis factor alpha (TNF-α), inflammatory cytokines (e.g., interleukin (IL)-6, IL-18)and ROS damage induce VSMCs to switch to a dedifferentiated synthetic phenotype [2,3,7] (Figure 1). The dedifferentiated synthetic VSMCs are epithelioid-shaped, expressing decreased levels of contractile proteins and increased levels of synthetic proteins (e.g., osteopontin (OPN), vimentin, S100 calcium-binding protein A4(S100A4), and N-myristoyltransferase 1 (NMT1)), and they exhibit a high proliferation and migration capacity and synthesize massive amounts of extracellular matrix (ECM) [1,3].

Usually, VSMCs phenotypic switching was limited to a transition between “contractual” and “synthetic” states, i.e., they switch to a synthetic state in response to various stimuli and then return to a contractile state after cessation of the stimuli. Recent studies have revealed that the differentiation and dedifferentiation of VSMCs is a multifactored process in which VSMCs can take on the features of other cell types, such as osteoblasts, chondrocytes, adipocytes, and macrophage foam cells [8]. For example, inflammatory stimulation or lipid accumulation may lead VSMCs to switch to macrophage-like cells, which begin to acquire macrophage and mesenchymal stem cell (SMC) markers, and display phagocytic activity. Then they phagocytose lipids and ultimately transform into foam cells [9,10].

VSMCs phenotypic transformation is mainly regulated by gene transcription, epigenetic modification, and signal transduction. At the gene transcription level, the key to VSMCs phenotypic regulation lies in the serum response factor (SRF), which binds to the CArG box sequence (*CC[a/T]6CC*) in muscle and cytoskeletal genes [11]. Myocardin is the most efficient known VSMCs differentiation driver SRF coactivator. CArG-SRF-myocardin complex formation is key to maintaining VSMCs differentiation [12]. Once the CArG-SRF-myocardin complex is disrupted, the regulation of the dedifferentiation/synthesis phenotype of VSMCs occurs. Other factors that play an important regulatory role at the gene transcription level include Krüppel-like factor 4 (KLF4), Forkhead box O 4 (FOXO4), and the transcriptional factor Elk-1 [13,14].

In terms of epigenetics, Ten-eleven-translocation 2 (TET2) acts upstream of MYOCD/SRF and KLF4 to influence phenotypic regulation by affecting chromatin accessibility at contractile and synthetic gene promoters. MicroRNAs often bind to the 3′ UTR and inhibit target gene translation, influencing phenotypic transformation through posttranscriptional regulation. MiR-143/-145, miR-133, miR-124, miR-370, miR-21, miR-24, and miR-29a can drive the transition of VSMCs to a differentiated phenotype [15,16]. MiR-206, miR-221, and miR-222 can drive the transition of VSMCs to a dedifferentiated contractile phenotype [17,18]. Recent studies have found that some non-coding RNAs, such as circMAP3K5, circDcbld1, and circ Lrp6, also modulate the VSMCs phenotypic transformation [19,20,21].

There are many other signaling pathways involved in the phenotypic transformation of VSMCs; for example, Ras/Raf/MEK/ERK, GSK3β, and β-actin can promote phenotypic transformation, and Rho-actin, TGF-β, andPI3K/Akt/mTOR can inhibit phenotypic transformation [9]. K-Cl cotransport (KCC) maintains cell volume and ion homeostasis in growth and differentiation, and several pieces of evidence indicate that electroneutral KCC is important for VSMCs proliferation, migration, and vasodilation [9,22].

## 2. Cardiovascular Diseases Associated with VSMCs Phenotypic Switching

### 2.1. Atherosclerosis

Atherosclerosis is a chronic inflammatory condition accompanied by the development of plaques and the narrowing of the vascular lumen [23]. Although atherosclerosis was initially thought to be a problem that plagued people concentrated in developed countries, it has become apparent that it is a global matter. With the increasingly effective prevention and control measures for infectious diseases, the improvement of health services, and better access to medical care, people now have a longer life expectancy, and they are more likely to be exposed to the adverse consequences of atherosclerosis [24,25], such as myocardial infarction due to thrombus formation when a plaque ruptures or erosion blocks the blood flow [26]. Although middle- to older-aged people are the primary population affected, an increasing number of younger women are now at risk, and an increasing proportion of patients of advanced age are women [27,28]. Endothelial lesions, cholesterol deposition, inflammatory cell invasion, foam cell creation and migration, and fibrous cap formation are all signs of atherosclerosis [29,30]. To date, the role of cholesterol-rich low-density lipoproteins (LDL) in atherosclerosis has been the most intensively and extensively studied. Recently, in addition to continued research on LDL, researchers have concentrated on HDL’s protective role and the altered behavior of VSMCs in the arterial wall and have linked it to inflammation and other risk factors such as sleep disorders, microbiota, and environmental stress [31].

During the lesion process, VSMCs are stimulated by the surrounding environment (TNF-α, PDGF-BB, ox-LDL, etc.); VSMCs utilize their plasticity to convert from a contraction to a synthesis phenotype or transdifferentiate into other cell types. In recent genealogical tracing in a mouse model of atherosclerosis, it was found that 70% of the cellular composition of atherosclerotic plaque lesions was VSMCs transdifferentiated into macrophage-like cells and osteochondrocytes [32]. VSMCs migrate to the endothelium to secrete extracellular matrix and participate in the formation of the fibrous cap due to their higher proliferation and migratory capabilities [5,33,34].

The impact of wall pressure on endothelial cells is well documented, whereas the impact of overlapping normal or pathological physical stressors on the intimal VSMCs is less well studied. The mix of hypertensive pressure and matrix adherence, as well as the prevalence of mechanical stimuli during atherosclerosis, leads to an intact VSMCs phenotype transition [35], according to Pamela Swiatlowska et al.

Galectin-1 (Gal-1) is a galactoside-binding protein that may serve as a new target for the treatment of atherosclerosis. Mice deficient in Gal-1 (Lgals1^−/−^) develop severe atherosclerosis with increased lipid levels in plaques and reduced expression of contractile markers in VSMCs compared to wild-type mice [36] (Table 1). The plaque volume is reduced, intraplaque oxidized low-density lipoprotein (ox-LDL) was significantly reduced, and the phenotypic transformation of VSMCs was suppressed in plaque-prone apolipoprotein E and CRP4 double knockout (dKO) mice [37].

VSMCs undergo a complex phenotypic during the progression of atherosclerotic disease, and single-cell sequencing of atherosclerotic aortic-derived CD45- cells from ApoE-deficient (ApoE^−/−^) mice fed a normal cholesterol diet (NCD) or high cholesterol diet (HCD) revealed that growth differentiation factor 10 (GDF10) mediates VSMCs osteogenic transition and is detrimental to atherosclerotic plaque stabilization [38].

Chronic kidney disease (CKD) is linked to faster atherosclerosis progression and a higher risk of cardiovascular incidents. CKD-induced oxidative stress triggers an increased IFN-I response in VSMCs, which causes premature VSMCs aging process and phenotypic switching in an autocrine/paracrine way, leading to fibrous cap VSMCs loss and fibrous cap pinching [39].

The inflammatory response and phenotypic transition of VSMCs were revealed in high-fat diet (HFD)-fed atherosclerosis in ApoE^−/−^ mice co-localized with atherosclerotic lesions and accompanied by elevated serum CDK9, and CDK9 knockdown reversed the ox-LDL-induced inflammatory response and phenotypic transition of VSMCs from contractile to synthesis phenotype by hindering the NF-κB pathway [40]. A new long-stranded non-coding RNA (lncRNA), PEBP1P2, can curb the growth and migration of VSMCs by directly binding to CDK9 [5]. DJ-1 deficiency leads to VSMCs phenotype switching and is dependent on KLF4 to exacerbate atherosclerotic plaque instability [24].

BMAL1 plays an important role in regulating circadian rhythms and is also an important regulator of atherosclerosis. BMAL1 is found at higher levels in unstable plaques in humans, accompanied by elevated YAP1 and the fibroblast marker FSP1. BMAL1 overexpression promotes the conversion of VSMCs to fibroblast-like cells by transcriptionally upregulating the expression of YAP1 [41]. A traditional Chinese medicine previously used to treat itchy skin, vertigo, and certain cardiovascular diseases, Furostanol saponins enriched extract (FSEE) of *T. terrestris* L., has been shown to inhibit the development of atherosclerosis and suppress the proliferation of VSMCs by inhibiting Akt/MEK/ERK signaling [42].

### 2.2. Intimal Hyperplasia and Restenosis

In healthy mature vessels, the intimal layer is composed mainly of endothelial cells, while the amount of contractile VSMCs in the intimal layer is negligible [4]. The arterial endothelium forms a monolayer of cells that covers the interior luminal side of vascular vessels and provides a barrier between blood and tissues. It is necessary to maintain vascular homeostasis and control the vasomotor tone [43,44].

As the endothelium suffers damage, the subendothelial collagen then becomes exposed, followed by a large amount of platelet adhesion and aggregation, while various inflammatory factors converge to the damaged endothelium, and then the activated VSMCs undergo dedifferentiation, proliferation and gradually migrate to the endothelium, where they secrete massive extracellular matrix [45,46,47]. The end result is the endothelial cells regenerate the covering over the intima, and the neointimal is formed in this manner [48]. It has been widely accepted that VSMCs, migration, and phenotype switching, as well as inflammation, are key events in the pathogenesis of vascular intimal hyperplasia [45,49,50,51].

Intimal hyperplasia (INH) is a normal complication of artery remodeling following a vascular injury [4,50,52]. Unfortunately, the uncontrolled proliferation of VSMCs and the deposition of vast amounts of extracellular matrix (ECM) will lead to excessive intimal hyperplasia and the consequent narrowing of the lumen, ultimately causing associated vascular diseases such as hypertension and atherosclerosis [4,7,49]. It can also occur as a result of vascular surgery, resulting in vascular stenosis and the failure of revascularization operations or worsening of the initial condition [48,53].

Percutaneous transluminal coronary angioplasty with balloon inflation applied to patients with coronary artery stenosis began to come into vogue in the late 1970s [54]. With the gradual maturity of percutaneous transluminal angioplasty (PTA), its clinical utilization rate has increased, but restenosis is accompanied by problems that limit its long-term efficacy [4,55]. Bare-metal stents then began to be phased into clinical practice. Compared with percutaneous transluminal coronary angioplasty with a balloon, the implantation of bare metal stents increases the risk of restenosis at the lesion by increasing the damage to the vessel and increasing the risk of endothelial hyperplasia [55,56,57]. In order to address this problem, there has been more research on stent materials and drug-eluting stents have been created [54,58]. Antiproliferative medicines, namely the chemotherapeutic agent paclitaxel and the mTOR inhibitor sirolimus, are placed on drug-eluting stents, and angioplasty balloons enable vascular wall distribution in current attempts to attenuate IH [4]. However, the nonspecific antiproliferative effect of these drugs on VSMCs and ECs leads to the need for long-term antiplatelet therapy after stent deployment, so further studies of drugs specifically targeting VSMCs are necessary to achieve anti-endothelial proliferation [59]. These improvements in stent technology have resulted in improved safety and effectiveness in the treatment of coronary artery stenosis. However, increasing evidence suggests that these variations still do not completely eliminate the problem of in-stent restenosis.

The angiogenic factor AGGF1 is required for phenotypic transition, proliferation, migration, and cell cycle regulation of VSMCs, and recent studies have shown that AGGF1 co-acts with integrin α7, a receptor on VSMCs, to reduce the expression of synthetic markers in VSMCs and block neointima formation after vascular injury [60] (Table 2).

Platelets are activated at the damaged endothelium and then secrete platelet-derived microvesicles (PMVs) that promote VSMCs dedifferentiation by Src/Lamtor1/mTORC [46]. Bone morphogenetic protein (BMP)-2 has been shown to play a role in controlling VSMCs phenotypic switching and vascular disease progression, and lysine (K)-specific demethylase 1A (KDM1A) was proven to target BMP-2, inhibiting the dedifferentiation of VSMCs and mitigating neointima formation and collagen deposition after surgery [7].

Nesfatin-1 acts as a modifier of cardiovascular function, upregulate matrix metalloproteinase 2 (MMP-2) and MMP-9 promoter activity, downregulates the peroxisome proliferator-activated receptor γ (PPARγ) content, and enhances the activity in VSMCs, promoting VSMCs dedifferentiation and stimulating VSMCs proliferation and migration. Blocking MMP-2/9 or activating PPARγ curbed nesfatin-1-induced VSMCs proliferating and migrating [61]. An in vitro model of neointimal hyperplasia was generated using AngII-stimulated rat thoracic aortic smooth muscle cells (A10 cells), while an in vivo model of neointimal hyperplasia was developed 2 weeks following carotid balloon injury in SHR rats. The results showed that fisetin could reduce oxidative stress and dose-dependently inhibit VSMCs proliferation and migration by activating PPARγ-induced antioxidant paraoxonase 2 (PON2) expression to attenuate neointimal hyperplasia after endothelial injury [4,55].

TET2 has recently been reported to regulate VSMCs phenotypic transition, protect ECs from harmful stimuli, and suppress the inflammatory response in atherosclerosis. New experiments confirmed that the activated endothelial CD137 signaling pathway attenuates the content of TET2 in EC-derived exosomes and inhibits platelet-derived growth factor (PDGF)-BB-induced VSMCs phenotypic transition and neointima formation following carotid injury [62].

It was found that after endothelial injury of mouse femoral arteries with guidewires, 5-MTP enhanced endothelial cell proliferation at the injury site and maintained the expression levels of genes related to the differentiation phenotype of VSMCs by reducing activated p38 MAPK and NFκB-p65, revealing a new protective role of 5-MTP in restenosis [63]. In wire-injured femoral arteries, overexpression of miR-22 dramatically reduced the expression of its target genes MECP2 (methyl-CpG binding protein 2) and histone deacetylase 4, lowered VSMCs proliferation, and impeded neointima formation [64]. Circular RNAs work by isolating certain microRNAs to control gene expression. circMAP3K5 (human circular MAP3K5) has been discovered to isolate miR-22-3p, which inhibits TET2 expression and suppresses human coronary VSMCs proliferation. CircLrp6 impedes VSMCs migration, proliferation, and differentiation regulation mediated by miR-145. CircDcbld1 works with miR-145-3p to regulate VSMCs migration by upregulating neuropilin-1 (Nrp1) levels [19,20,21].

### 2.3. Aortic Aneurysms

An aortic aneurysm (AA) is a weakening of the aortic wall that results in progressive dilatation. There are two types of its AA based on anatomic location: thoracic aortic aneurysm (TAA) and abdominal aortic aneurysm (AAA) or thoraco-abdominal AA (Crawford classification) [65]. AA lacks symptoms and is usually detected during an ultrasound examination [66]. Hypertension, cholesterol, atherosclerosis, smoking, and male sex are all risk factors for AA, with smoking being the vitally significant controllable risk factor, and it has been shown that smoking cessation lowers the risk of acquiring AA and restricts the progression of already existing AAs [66,67].

There is still no reliable drug treatment for aneurysms. Currently, the only treatment for aortic aneurysms is surgery. In the case of a ruptured aneurysm, the patient may rapidly die if they do not undergo surgery in the timeliest manner. A considerable number of studies are being conducted to better understand the pathogenesis of AA and to find innovative and feasible means of prevention and treatment. Previous studies have suggested that VSMCs apoptosis is a critical part of atherogenesis, but according to recent studies, the VSMCs phenotype is altered before or early during the establishment of aneurysms [68]. Ailawadi and his team found that VSMCs contraction markers were downregulated and matrix metalloproteinases were upregulated early in aneurysmogenesis, suggesting that VSMCs phenotypic transformation occurs during this period. Matrix metalloproteinases destroy the elastin and collagen that make up the aortic wall, thereby destroying the strength of the aortic wall [69,70]. Therefore, maintaining VSMCs stability may be a potential target to inhibit aortic aneurysm formation or its early progression from the beginning.

LGMN (legumain) has been shown to induce the development of thoracic aortic dissection (TAD) by degrading the extracellular matrix, and Lihong Pan et al. found in angiotensin II and BAPN-induced thoracic aortic dissection (TAD) models that LGMN could downregulate VSMCs differentiation markers and exacerbate TAD development by binding to and blocking integrin αvβ3 in VSMCs and attenuating Rho GTPase activation [71] (Table 3).

CircChordc1 was found to be significantly deregulated in aneurysmal tissue compared to healthy arteries, and it was capable of converting VSMCs to a contractile phenotype and improving their growth by triggering waveform protein degeneration and boosting the GSK3/-catenin pathway, which inhibited aneurysm formation and minimized the chances of fracture in angiotensin II and CaCl2-induced AAA mouse models [72].

Insulin resistance (IR) or type 2 diabetes mellitus (T2DM) have previously been proven to be independent predictors for a variety of cardiovascular illnesses, but there have been few studies on the link between IR and aortic dissection (AD). Hui Zheng et al. found that IR induced a phenotypic shift in VSMCs from contractual to synthetic phenotypes, thereby promoting AD [78].

MiR-23b expression was observed by Si et al. to be considerably reduced in the aorta of angiotensin II-treated ApoE^−/−^ mice. In an in vitro test, knocking down miRNA-23b during an angiotensin II-induced VSMCs phenotypic transition greatly boosted the expression of the transcription factor Forkhead box O4 (FoxO4), which stabilizes the VSMCs contractile phenotype and protects against AAA formation [68].

Fc receptor (FcR) upregulation was found in the outer and middle membranes of human and mouse AAA, and FcR deficiency inhibited IC-triggered inflammatory gene expression, oxidative stress, phenotype switching in vitro, and AAA formation [73]. Suppressor of cytokine signaling pathway 1 (SOCS1), a negative regulator of the JAK/STAT signaling pathway, prevents the development of AAA by inhibiting STAT1/3 activation in the aorta, downregulating cytokines, metalloproteinases, and increasing the expression of differentiation markers in contractile VSMCs [74].

Anxa1 blocks the production of a synthesis phenotype of VSMCs by downregulating the JunB/MYL9 pathway, which leads to increased inflammation and enhanced matrix metalloproteinase (MMP) production, resulting in increased elastin degradation and subsequent worsening of acute aortic dissection (AAD) [75]. It was revealed that 5’-tiRNA-Cys-GCA was decreased in human and mouse aortic dissection models, and 5’-tiRNA-Cys-GCA overexpression inhibited the proliferation and migration of vascular smooth muscle cells through STAT4, upregulated contractile markers, reduced the incidence of angiotensin II and β-aminopropionitrile-induced aortic dissection (AD) in mice and prevented its deterioration [76]. NCOR1 (nuclear receptor corepressor1) interacts with FOXO3a, NFAT5, and ATF3 to maintain the contractile phenotype of VSMCs and inhibit AA development through three mechanisms [77].

### 2.4. Vascular Calcification

Atherosclerosis, hypertension, and aneurysms all cause vascular calcification, which is apparent in the medial and intimal walls of the vessels [79,80]. As mentioned earlier, the plasticity of VSMCs is not binary, and they can display characteristics of osteoblasts, adipocytes, chondrocytes, and macrophage foam cells [81]. Factors such as elevated calcium and phosphorus in blood could stimulate the conversion of VSMCs to osteogenic phenotypes, which possess calcium binding capacity and the ability to produce osteoblast-like ECM [82,83]. Intracellular calcium overloading occurs in VSMCs exposed to elevated calcium levels, starting with microcalcification and gradually leading to massive calcification and then to vascular stiffness [84,85,86,87].

Calcification is more common in the carotid plaques of smokers compared to nonsmokers, and the difference in microcalcifications is particularly significant, confirming a higher propensity for smokers to have a higher atherosclerotic burden. Nicotine increases osteogenic gene expression (Runx2, Osx, BSP, and OPN), thereby inducing primary VSMCs calcification in humans [88] (Table 4). In patients with chronic kidney disease (CKD), an endoplasmic reticulum (ER) stress-mediated phenotypic switch in vascular smooth muscle cells (VSMCs) is critical for vascular calcification (VC). Terpinen-4-ol hinders posttranslational PERK formation at the K889 acetylation site by increasing SIRT1 expression and improving VC by regulating the ER pressure [89]. Interleukin enhancer binding factor 3 (ILF3) expression was found to be increased in calcified human aortic vascular smooth muscle cells (HAVSMCs) and calcified atherosclerotic plaques in humans and mice, and ILF3 promoted atherosclerotic calcification by acting on the promoter regions of BMP2 and STAT1 and mediating BMP2 upregulation and STAT1 downregulation, according to Fei Xie et al. [90] BGP-15 is an emerging antidiabetic drug that has been found to inhibit Pi-induced phenotypic transformation during the osteochondrogenesis and mineralization of VSMCs, making it potentially ideal for reducing diabetic and nondiabetic vascular calcification [91]. In vitro experiments demonstrated that miR-223-3p blocked interleukin 6 (IL-6)/STAT3 signaling, thereby preventing osteogenic transformation and calcification in VSMCs [92]. During calcification of VSMCs, KMUP-3 inhibits mTOR and β-linked protein upregulation and enhances AMP-activated protein kinase (AMPK) activation, thereby preventing VSMCs phenotype switching [93].

## 3. Conclusions and Perspectives

VSMCs are vital and complex cells found in the vascular wall that are capable of undergoing reversible differentiation and dedifferentiation in response to various stimuli or switching into completely different cell types, accompanied by changes in proliferation, migration, and secretory function to perform physiological or pathological functions in specific settings. Phenotypic switching of VSMCs is a common feature in the pathogenesis of several cardiovascular diseases. At present, many studies are focusing on exploring the molecular mechanism of the phenotypic transformation of VSMCs in different diseases. In this paper, we summarize the most recent study results, which could facilitate the development of preventive and treatment strategies for these diseases. However, the origin of VSMCs that play a role in the disease process is often ambiguous, and how to determining the origin of pathological VSMCs is an urgent challenge to overcome. At what stage of the disease process VSMCs begin to undergo pathological changes, and what characteristic markers indicate the VSMCs are in a pathological state are also questions worth studying in the future, which should assist in distinguishing VSMCs subsets more precisely and help us take measures to prevent these diseases.

## Figures and Tables

**Table 1:** Molecular mechanisms of VSMCs phenotypic switching in atherosclerosis.

Protein/RNA	Animal Model		Downstream Molecule/ Signaling Pathway	Promotes (+)/Inhibits (−) VSMCs Phenotypic Switching	VSMCs Differentiation Markers	VSMCs Dedifferentiation/ Transdifferentiation Markers	Reference
Galectin-1	Mice	Severe atherosclerosis induced by pAAV/D377Y-mPCSK9 adenovirus		-	α-SMA		[36]
Cysteine-rich LIM-only protein 4 (CRP4)	Mice	ApoE^−/−^ mice fed with Western diet		+	α-SMA		[37]
Growth Differentiation Factor 10 (GDF10)	Mice	ApoE^−/−^ mice fed with high cholesterol diet (HCD)		+	α-SMA SMMHC	Alkaline Phosphatase RUNX2	[38]
Type-I-interferon (IFN-I)	Mice	ApoE^−/−^ mice		+	α-SMA SMMHC CNN1		[39]
Cyclin-dependent kinases 9 (CDK9)	Mice	ApoE^−/−^ mice fed with high fat diet (HFD)	NF-κB	+	α-SMA	Vimentin OPN	[40]
PEBP1P2	Rats	Rats carotid artery injury models	CDK9	−	α-SMA SMMHC CNN1		[5]
Deglycase DJ-1	Mice	ApoE^−/−^ mice fed with western diet	KLF4	−	α-SMA CNN1	Vimentin OPN	[24]
BMAL1	Mice	Severe atherosclerosis induced by pAAV/D377Y-mPCSK9 adenovirus, western diet	YAP1	+	SMMHC		[41]
Furostanol saponins enriched extract (FSEE) of *T. terrestris* L.	Mice	ApoE^−/−^ mice fed with high fat diet (HFD)	Akt/MEK/ERK	−	α-SMA	OPN	[42]

α-SMA: α-smooth muscle actin. SMMHC: smooth muscle myosin heavy chain. CNN1: calponin 1. OPN: osteopontin. NF-κb: nuclear factor kappa-B. CDK9: cyclin-dependent kinases 9. KLF4: transcription factor 4. YAP1: yes1 associated transcriptional regulator. Akt: protein kinase B. MEK: MAPK/ERK kinase. ERK: extracellular regulated protein kinases.

**Table 2:** Molecular mechanisms of VSMCs phenotypic switching in Neointimal formation.

Protein/RNA	Animal Model		Downstream Molecule/ Signaling Pathway	Promotes (+)/Inhibits (−) VSMCs Phenotypic Switching	VSMCs Differentiation Markers	VSMCs Dedifferentiation/ Transdifferentiation Markers	Reference
Angiogenic factor AGGF1	Mice	Mice carotid artery wire injury model	Integrin α7	+	α-SMA SMMHC SM22α		[60]
Nesfatin-1	Rats	Rats carotid artery injury	MMP2/MMP-9 PPARγ	+	α-SMA SMMHC	OPN	[61]
CD137	Mice	Mice carotid artery wire injury model	TET2	−	α-SMA SMMHC CNN1	Vimentin	[62]
Tryptophan metabolite 5-methoxytryptophan (5-MTP)	Mice	Mice femoral artery denudation injury model	p38 MAPK NFκB-p65	−	α-SMA		[63]
CircRNA_009723 (circDcbld1)	Mice	Common carotid artery (CCA) intima hyperplasia model	miR-145-3p/Nrp1	+	α-SMA SMMHC CNN1		[20]
Lrp6 (lipoprotein receptor 6)	Mice	ApoE^−/−^ mice placed in perivascular collar	miR-145	−	α-SMA SM22α		[21]
Circular mitogen-activated protein kinase 5 (CircMAP3K5)	Mice	Mice femoral artery wire denudation model	miR-22-3p/TET2	+			[19]
Lysine (K)-specific demethylase 1A (KDM1A)	Rats	Rats carotid artery balloon injury model	BMP-2	+	α-SMA	OPN	[7]
Platelet-derived microvesicles (PMVs)	Mice	Mice carotid wire injury model	Src/Lamtor1/mTORC1	+	α-SMA SM22α CNN1		[46]
FISETIN	Rats	Rats carotid artery balloon injury model	PPARγ/PON2	−	α-SMA	OPN	[55]

α-SMA: α-smooth muscle actin. SMMHC: smooth muscle myosin heavy chain. SM22α: smooth muscle protein 22-α. CNN1: calponin 1. OPN: osteopontin. NFκB: nuclear factor kappa-B. MMP: matrix metallopeptidase. PPARγ: peroxisome proliferator activated receptor γ. TET2: tet methylcytosine dioxygenase 2. MAPK: mitogen-activated protein kinase. Nrp1: neuropilin 1. mTOR: mammalian target of rapamycin. PON2: paraoxonase 2.

**Table 3:** Molecular mechanisms of VSMCs phenotypic switching in Aortic aneurysm.

Protein/RNA	Animal Model		Downstream Molecule/ Signaling Pathway	Promotes (+)/ Inhibits (−) VSMCs Phenotypic Switching	VSMCs Differentiation Markers	VSMCs Dedifferentiation/ Transdifferentiation Markers	Reference
Legumain	Mice	Mice model of BAPN-induced TAD	Integrin αvβ3	+	SM22α SMMHC CNN1		[71]
Cysteine and histidine-rich domain containing 1 (CircChordc1)	Mice	Mice model of angiotensin (Ang) II- and CaCl2-induced AAA	Annexin A2 and glycogen synthase kinase 3 beta (GSK3β)	−	SM22α CNN1	Vimentin OPN	[72]
MicroRNA-23b	Mice	Mice model of AngII-induced AAA	FoxO4	−	α-SMA SM22α CNN1		[68]
Fcγ receptor	Mice	Mice model of aortic perfusion of elastase from porcine pancreas induced AAA		+	α-SMA SM22α	KLF4	[73]
Cytokine signaling-1 (SOCS1)	Mice	Mice model of elastase-induced AAA	JAK/STAT	−	α-SMA SM22α CNN1	KLF4	[74]
Anxa1	Mice	Mice model of AngII-induced AAD	JunB/MYL9	−	α-SMA SMMHC SM22α CNN1		[75]
5’-tiRNA-Cys-GCA	Mice	Mice model of intraperitoneal injection of AngII combined with intraperitoneal injection of BAPN induced AAA	STAT4	−	α-SMA SMMHC CNN1		[76]
Nuclear receptor corepressor1 (NCOR1)	Mice	Mice model of angiotensin (Ang) II	FoxO3a NFAT5 ATF3	−	α-SMA SMMHC CNN1		[77]

α-SMA: α-smooth muscle actin. SMMHC: smooth muscle myosin heavy chain. SM22α: smooth muscle protein 22-α. CNN1: calponin 1. OPN: osteopontin. JAK: janus kinase. STAT: signal transducer and activator of transcription. JunB: jun. B proto-oncogene. MYL9: myosin light chain 9. FoxO3a: forkhead box o3a. FoxO4: forkhead box o4. NFAT5: nuclear factor of activated t cells 5. ATF3: activating transcription factor 3. KLF4: krüppel-like factor 4. TAD: thoracic aortic dissection. AAA: abdominal aortic aneurysm. AAD: acute aortic dissection.

**Table 4:** Molecular mechanisms of VSMCs phenotypic switching in Vascular calcification.

Protein/RNA	Animal Model		Downstream Molecule/ Signaling Pathway	Promotes (+)/Inhibits (−) VSMCs Phenotypic Switching	VSMCs Differentiation Markers	VSMCs Dedifferentiation/ Transdifferentiation Markers	Reference
Nicotine			Nox5	+	α-SMA SM22α CNN1	S100A4 KLF4	[88]
Terpinen-4-ol	Mice	Mice fed with a high phosphorus diet supplemented with adenine	Sirtuin 1 (SIRT1)	−	α-SMA	RUNX2 ALP BMP2	[89]
Interleukin enhancer Binding factor 3 (ILF3)	Mice	ApoE^−/−^ mice fed with high fat diet (HFD)	BMP2 STAT1	+	α-SMA	BMP2 RUNX2 STAT1	[90]
BGP-15			Annexin A2	−	α-SMA	KLF-5 Msx-2 Sp7 BMP-2	[91]
KMUP-3	Mice	Mice model of AngII-induced AAA	AMPK	−	α-SMA	RUNX2	[93]

α-SMA: α-smooth muscle actin. SM22α: smooth muscle protein 22-α. CNN1: calponin 1. S100A4: S100 calcium binding protein A4. Nox5: NADPH Oxidase 5. BMP2: bone morphogenetic protein-2. STAT1: signal transducer and activator of transcription 1. AMPK: adenosine 5‘-monophosphate (AMP)-activated protein kinase. KLF: krüppel-like factor. RUNX2: RUNX family transcription factor 2. ALP: alkaline phosphatase. Msx-2: msh homeobox 2. Sp7: Sp7 transcription factor.
